# Rearing *C. elegans* on Parafilm-wrapped NGM Plates Impacts Habituation Behavior

**DOI:** 10.17912/micropub.biology.000760

**Published:** 2023-03-31

**Authors:** Jessica Chalissery, Isabel Wilson, Catharine Rankin, Joseph Liang

**Affiliations:** 1 Djavad Mowafaghian Centre for Brain Health, University of British Columbia, Vancouver, British Columbia, Canada; 2 Department of Psychology, University of British Columbia, Vancouver, British Columbia, Canada

## Abstract

Scientists use Parafilm to seal *Caenorhabditis elegans* cultures on Nematode Growth Media (NGM) petri plates for short-term storage to reduce the likelihood of contamination and improve moisture retention. However, we found that maintaining worms on plates wrapped with Parafilm can affect multiple behavioral metrics when assaying tap-habituation behavior using the Multi-Worm Tracker (MWT). Most notably, worms cultured on parafilm-wrapped NGM plates exhibited slower speed of initial response to tap followed by marked sensitization. These findings suggest that labs should be conscious of the possibility that Parafilm may induce behavioral changes in *C. elegans* when conducting experiments.

**Figure 1. Comparison of habituation behavior in Caenorhabditis elegans reared on Parafilm-wrapped vs non-wrapped plates.**
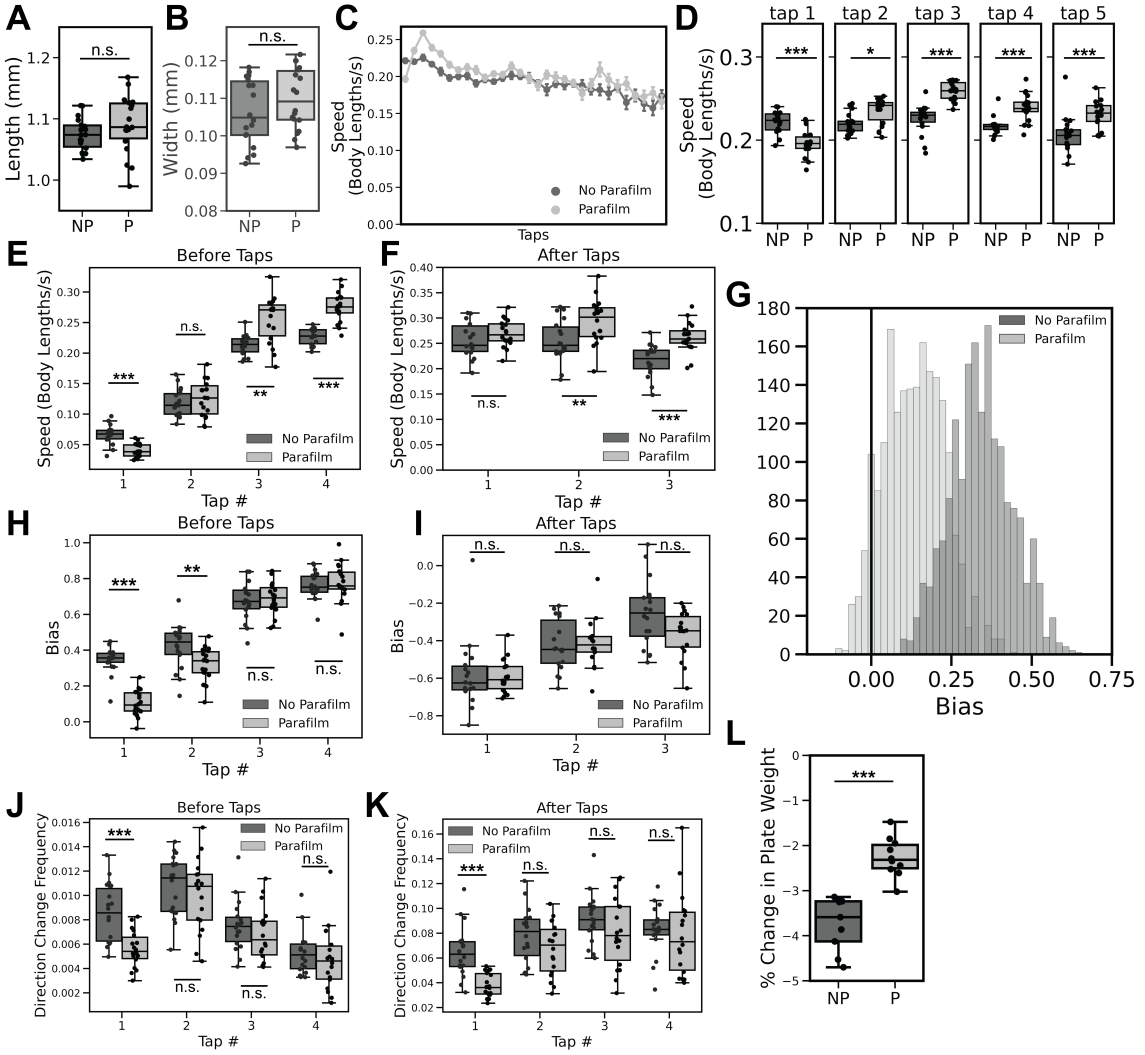
In all panels, the Parafilm-wrapped (P) condition is represented by light grey and the non-wrapped (NP) condition is presented in dark grey. All worms are N2 wild-type, fed with OP50 *E. coli* , tested at age 96h. Worm length **(A)** and width **(B)** were not significantly different between the two conditions. **(C)** Habituation of speed of reversal of both Parafilm-wrapped and non-wrapped worms, measured in body lengths/s over the course of 30 taps. Error bars denote standard error of the mean. **(D)** Reversal response speed to only the first five stimuli were statistically different between groups. **(E)** Speed of movement before the first four taps was measured in the interval beginning 3s before the tap and ending 0.1s before the tap. **(F)** Speed of movement after the first four taps was measured in the interval beginning 0.1s after the tap and ending 1s after the tap. **(G)** Population frequency distributions of the bias measure for the two conditions, where an increasing positive value on the X-axis indicates an inclination for forward motion, and an increasingly negative value indicates more backward motion in locomotion behavior. Bias before the first four taps **(H)** and after the first three taps **(I)** , and the frequency of direction change before **(J)** and after **(K)** the first four taps were also compared between the two conditions. **(L)** The amount of weight loss in the plates was compared between the two conditions after 11 days. * = p<0.05 ** = p<0.01 *** = p<0.001.

## Description

With the advancements in machine vision and artificial intelligence to facilitate the process of analyzing the behaviors of freely crawling animals, *C. elegans* scientists can employ tracking systems to perform high-throughput screens to characterize a broad range of behavioral phenotypes (McDiarmid et al., 2018). Our lab developed one such tracking system, the Multi-Worm Tracker (Swierczek et al., 2011), which is capable of analyzing morphology and tracking numerous metrics of behavior for more than 100 free-living animals simultaneously, in real-time. One well-studied behavior in *C. elegans* is habituation of the tap withdrawal response in which animals crawl backward for a short distance in response to a mechanical tap to the agar-filled plate on which the worms are reared (Rankin, 2002; Giles & Rankin, 2009; Bozorgmehr et al., 2013; Chen & Chalfie, 2014). With each repeated tap stimulus, this reversal response declines. Recent work has shown that there may be multiple genetic mechanisms underlying habituation to tap as different components of the response (i.e. probability, duration or speed of the response) show different patterns of response decrement and seem to be altered by mutations in different genes (McDiarmid et al., 2019).

In the lab, nuanced differences in tap-habituation profiles of adult wild-type populations collected by different lab members suggested that slight differences in protocols were altering what should be a consistent phenotype. A common practice in *C. elegans* labs that maintain populations on solid media is to use Parafilm, a flexible, semi-transparent thermoplastic, to seal the nematode growth medium (NGM) plates upon which the worms are cultured to avoid bacterial contamination and retain moisture. A key and consistent variation in protocols was whether lab members used Parafilm to wrap NGM plates, versus leaving the plates unwrapped after age-synchronizing the population 96 hours before they were to be tracked. To date, relatively little has been done to investigate the effects of parafilm on nematode behavior and physiology. Shinn-Thomas et al. (2019) found that the use of Parafilm to wrap NGM plates had a significant effect on the growth of the worms compared to plates that were not wrapped, up to 48 hours post-hatch (through larval development). Petri plates are designed to allow for free movement of gas and moisture; Thus, the use of Parafilm to seal the plates may affect a variety of factors in the environment the worms are cultured in, such as temperature, humidity, and the level of oxygen available within the plate. If the use of Parafilm affects behavior in *C. elegans* , it is important to standardize the procedures for its use so that comparisons can be made between data generated by individual lab members.

It was previously reported that larval worms reared on plates wrapped with Parafilm grew significantly faster compared to worms reared on non-wrapped plates when size was measured between hatching and 48 hours post-hatching (Shinn-Thomas et al., 2019). Because our worms were tested at 96 hours post-hatch, we extended this and measured adult worm length and width at 96 hours of age. Interestingly, Parafilm-wrapped plates did not yield significantly longer or wider worms compared to non-wrapped plates (Fig. 1A-B, Mann-Whitney U test, Worm Width U = 117.0, p > 0.05; Worm Length U = 120.0, p > 0.05). However, this is not surprising because by 96 hours (Day 4) animals would have reached the point of maturation where the rate of growth in size has significantly decreased (Croll et al., 1977).

Plates of worms in the two conditions (parafilm-sealed and not parafilm-sealed) were given 30 mechanical tap stimuli 10 seconds apart and habituation of response probability, duration and speed was analyzed. We found replicable differences between the two conditions for the speed of reversal responses to repeated tap stimuli and not for other measures (Fig. 1C). Compared to worms reared without Parafilm, worms reared on Parafilm-sealed plates exhibited slower initial speed of response to tap, which then increased before adopting the stereotypical habituation phenotype through the rest of the experimental paradigm (Fig. 1C). This is a sensitization phenotype not seen in the control group, which showed a steady decrease or habituation of the speed of response (Fig. 1C). Speed of response to the initial tap in the Parafilm condition was significantly lower than the initial response in the control condition (Fig. 1D; Mann-Whitney U test, Tap 1 U = 285.0, p < 0.001), but was significantly higher to the next 4 taps (Fig. 1D; Mann-Whitney U test, Tap 2 U = 81.0, p < 0.05; Tap 3 U = 8.0, p < 0.001; Tap 4 U = 40.0, p < 0.001; Tap 5 U = 52.0, p < 0.001), displaying a clear sensitization phenotype. After the 10th tap there were no differences in reversal speed throughout the rest of the experiment and worms in both conditions habituated to similar speed levels (Fig. 1C).

Outside of habituation of speed of responses to tap, we discovered differences in several other behavioral measures. Analysis of the speed of worms before taps (defined as the locomotor speed of animals between 3s – 0.1s prior to the tap stimulus), yielded a significant difference between the two conditions for the first, third, and fourth taps (Fig. 1E; Mann-Whitney U test, Tap 1 U = 297.0, p <0.001; Tap 2 U = 142.0; p > 0.05; Tap 3 U = 61.0, p < 0.01; Tap 4 U = 11.0, p < 0.001). There was also a significant difference in speed between the second and third taps during the interval 0.1s – 1s after the taps occurred (Fig. 1F; Mann-Whitney U test, Tap 1 U = 124.0, p > 0.05; Tap 2 U = 80.0, p < 0.01; Tap 3 U = 39.0, p < 0.001). Together, these results show that there was a difference in locomotor speed prior to the initiation of tap stimuli, and that difference was also present prior to the delivery of the first few taps.

Speed is not the only behavioral measure affected by the use of parafilm; bias (an indicator of preferred direction of movement) and frequency of direction change are baseline behaviors quantifiable by the MWT. We observed a distinct difference in bias, where a more positive value indicates a propensity for forward motion and a more negative value indicates more time spent reversing. Worms reared on plates wrapped in Parafilm displayed a lower amount of overall forward motion compared to worms reared on non-wrapped plates at baseline (Fig. 1G). Additionally, we analyzed bias before and after each tap stimulus. Before the first and second taps, bias was significantly lower (less forward movement) in the parafilm condition compared to the control condition (Fig. 1H; Mann Whitney U test, Tap 1 U = 316.0, p < 0.001; Tap 2 U = 246.0, p < 0.01), but there were no differences for any of the following taps (Mann Whitney U test, Tap 3 U = 148.0, p > 0.05; Tap 4 U = 146.0, p > 0.05). There were no significant differences in bias between the two conditions after individual taps (Fig. 1I; Mann Whitney U test, Tap 1 U = 156.0, p > 0.05; Tap 2 U = 154.0, p > 0.05; Tap 3 U = 216.0, p > 0.05). This was not surprising, as populations largely adopt the stereotypical reversal response to the tap-stimulus, and as mentioned previously, we did not observe a difference in response probability between the two groups.

In the Parafilm condition, the frequency of direction changes before taps was significantly lower than the non-Parafilm condition only for the first tap (Fig. 1J; Mann-Whitney U test, Tap 1 U = 278.5, p < 0.001; Tap 2 U = 188.0, p > 0.05; Tap 3 U = 192.0, p > 0.05; Tap 4 U = 193.0, p > 0.05), showing that at baseline, worms reared on Parafilm-wrapped plates make fewer spontaneous reversals than worms reared on non-wrapped plates. Similarly, the magnitude of direction changes after taps occur is only significantly different between conditions for the first tap (Fig. 1K; Mann-Whitney U test, Tap 1 U = 295.0, p < 0.001; Tap 2 U = 188.0, p > 0.05; Tap 3 U = 197.0, p > 0.05; Tap 4 U = 184.0, p > 0.05). As is the case for bias, frequency of direction change shifts to a comparable level in both conditions.

One potential causal difference between the Parafilm and No Parafilm conditions was the loss of water content of the agar in the NGM plate due to evaporation. Over the duration of plate storage, this can lead to drier plates with increasing age. We compared the amount of weight lost in plates in each condition over the course of 11 days – from the day the agar was poured into the NGM plates, to the day that they would have been used for testing – by measuring the decrease in weight of the NGM plates over time. Plates in the Parafilm condition lost significantly less weight than plates in the non-Parafilm condition (Fig. 1L; Mann-Whitney U Test, U = 0, p < 0.001).

Here, our data show that rearing worms in a Parafilm-wrapped environment can cause differences in baseline and habituation behavior. Animals reared on Parafilm-wrapped plates exhibited a significantly slower response speed to the first tap stimulus compared to non-wrapped plates, followed by what appears to be an increase in response speed in an apparent sensitization effect (Fig. 1C, D). Of the response metrics analyzed, we only saw differences between the two conditions in the habituation of the speed of response to tap. This is in line with our previous observations that parameters of habituation do not share common molecular mechanisms (McDiarmid et al., 2019). It was initially hypothesized that a difference in size may be an explanation for the varying behavior in the two conditions; However, contrary to the findings of Shinn-Thomas et al. (2019), we found that worms reared on Parafilm-wrapped plates were not larger than worms from non-wrapped plates at 96 hours of age (Fig. 1A, B). It is possible that parafilm might induce a developmental delay impacting the size of animals by 48 hours of age, but as worms continue to grow and the overall growth rate slows the effect is no longer apparent by 4 days old (96 hours).

In addition to loss of moisture in the NGM plates (Fig. 1L), we also considered other possible environmental factors that may have been impacted by the use of Parafilm including oxygen levels, temperature, and humidity. *C. elegans* have a hypoxia pathway that is activated when there is a lack of oxygen, which would result in a reduced metabolic rate (Cheung et al., 2005). However, this pathway is only activated when oxygen levels are below 1%; *C. elegans* are able to adapt fairly well when levels are between 1-21% (Cheung et al., 2005). Because Parafilm is permeable to oxygen, it is highly unlikely that the level of oxygen dropped to a point that would result in the hypoxia pathway being activated even if it causes any differences in oxygen levels within the plate. Large changes in external temperature can cause fluctuations in metabolic rate and subsequently behavior, but small changes in temperature (less than 5 degrees) do not yield significant behavioral changes (Zhao et al., 2003). As the worms were raised in temperature-controlled laboratory conditions (kept consistently at 20℃), it is unlikely that large fluctuations in temperature would have occurred, even with Parafilm present. Previous studies showed that high levels of humidity can cause worms to perform more reversals (backward locomotion; Parida & Padmanabhan, 2016; Zhao et al., 2003). It is possible that Parafilm increases the ambient humidity of the plate, increasing the number of active worms which resulted in sensitization (increased speed of reversals in response to tap stimuli) during the initial phase of the habituation paradigm. The difference in weight loss between wrapped and non-wrapped plates (Fig. 1L) reflects a loss of moisture in the agar of the NGM plates, which may play an important role in causing the phenotypes observed in the present study.

It is important to note that worms in the Parafilm-wrapped group also experienced a new environment when they were unwrapped for testing (plates were unwrapped for testing to prevent the addition of Parafilm from dampening the physical tap stimuli), as wrapped plates would have an environment distinct from that of an unwrapped plate. In contrast, worms from non-wrapped plates would not have experienced a change in environment. The Parafilm condition yielded worms that were significantly different in speed, bias and frequency of direction change from non-wrapped worms before the first tap stimuli occurred (Fig. 1E, H, J), indicating that there was a difference in baseline behavioral states between these two conditions. The observed behavioral differences did not persist very long in the experiment (Fig. 1C, E, F, H-K) suggesting the possibility that Parafilm-wrapped animals habituated to both the repeated tap-stimulus and the changed environment over time. Overall, these results show that using Parafilm during culture for *C. elegans* behavioral studies may alter consequent behavior, and that even the smallest of differences in protocol can alter behavior, emphasizing the importance of standardizing procedures throughout experiments both within and between labs.

## Methods

**Strain Maintenance and Behavioral Tracking**

The N2 wild-type *C. elegans* strain was maintained at 20℃ on Nematode Growth Medium (NGM) agar petri plates. Populations were age-synchronized on plates seeded 24 hours prior with 50uL of OP50 *E. coli* , spread evenly onto the entire area. Age-synchronized populations of roughly 100 of worms per plate were reared in a dark, mechanosensory-deprived, and temperature-regulated environment at 20℃ for 96 hours before tracking on the MWT. Nine plates were wrapped with Parafilm, and as a control group, nine plates were left unwrapped (Fig. 1A). Parafilm strips were cut from a 4in. x 250ft. Parafilm M roll roughly 2cm strips lengthwise (4in. by 2cm), and parafilm-wrapped plates were wrapped with single parafilm strips on average twice around the circumference of the plate, which expends the entirety of the strip. At the time of testing worms were unwrapped, then immediately tested on the MWT (McDiarmid et al., 2018) using a short-term mechanosensory habituation paradigm in which 30 tap stimuli were administered with a 10-second ISI after a 600-second acclimatization period. Data presented in this study was collected from two replicate experiments conducted on two separate days. All plates were unwrapped for testing to prevent Parafilm from dampening the physical tap stimuli. Worms were tracked at the age of 96 hours, at 20℃ and 40% humidity. To determine moisture loss of parafilm-wrapped versus non-wrapped plates, the plates were poured, kept in a temperature and humidity-controlled environment and weighed 11 days later, on the day they would typically be used for testing.

**Statistical Analyses and Data Visualization**

The Multi-Worm Tracker software (version 1.2.02; Swierczek et al., 2011) was used for automated stimulus delivery and real-time digital image acquisition and phenotyping. Tap-response and baseline phenotypic quantification were performed with custom open-source java software Choreography.jar using built-in filters to restrict analyses to animals that moved at least 2 body lengths and were tracked for at least 20s. A complete description of morphology, baseline locomotion and habituation learning features can be found in the Multi-Worm Tracker user guide ( https://sourceforge.net/projects/mwt/ ; Swierczek et al., 2011). To measure behavioral responses to tap-stimuli, the ‘MeasureReversal’ plugin was used to identify reversal behaviors that occurred within 1s of the mechanosensory stimulus onset. Custom python scripts were written on Jupyter notebook with seaborn 0.12.01 and pingouin 0.5.2 packages and their main dependencies to organize, summarize, analyze, and visualize data from Choreography output files. All features were pooled across plate replicates for each treatment, and the distributions of the two conditions were compared with the Mann-Whitney U test.

## Reagents

**Table:** 

Strain	Genotype	Available From
N2	Caenorhabditis elegans	CGC
OP50	Escherichia coli	CGC

Lab Recipe for 1L Nematode Growth Medium (NGM), for which 12mL was dispensed per plate to produce agar plates for MWT experiments:

**Table:** 

Reagent	Notes	Quantity	Source	Catalogue #
Agar		17.0g	Milipore Sigma	A7002
Gibco ^TM^ Bacto-peptone		2.48g	Fisher	DF0118170
NaCl		5.0g	Milipore Sigma	S9888
1M MgSO _4_	Dissolved in dH2O	1.0mL	Fisher	M65500
1M CaCl _2_	Dissolved in dH2O	1.0mL	Fisher	C79500
12.93mM (5mg/mL) Cholesterol	Dissolved in ETOH	1.0mL	Milipore Sigma	C8503
1M KH2PO4	Dissolved in dH2O	25.0mL	Fisher	BP362
dH _2_ O		1.0L		

**Other Materials**

**Table:** 

Item	Source	Catalogue #
MWT plates: Fisherbrand ^TM^ Drosophila Supplies Petri Dish	Fisher	AS4051
Parafilm-M, Benis 10.2cm (4”) x 76.2m (250’)	VWR	52858-032
